# Informational continuity by midwives during birth at primary care settings in the Western Cape

**DOI:** 10.4102/hsag.v29i0.2432

**Published:** 2024-04-29

**Authors:** Victoria J. Anthony, Anneline E. Robertson, Doreen K.M. Kaura

**Affiliations:** 1 Department of Nursing and Midwifery, Faculty of Medicine and Health Sciences, Stellenbosch University, Cape Town, South Africa

**Keywords:** continuity of care, care coordination, intrapartum period, midwifery led unit, primary health care, informational continuity

## Abstract

**Background:**

Informational continuity ensures that all health and psychosocial information of the pregnant women is available at all encounters with healthcare providers. The World Health Organization recognised that ineffective informational continuity during birth contributed to fragmented care and duplication of services, which ultimately influenced the morbidity and mortality rates of the pregnant women.

**Aim:**

The aim of this study was to delve into the midwives’ experiences on informational continuity approaches that enable effective care coordination during birth within the primary health care setting.

**Setting:**

The study setting was two maternity obstetric units (MOUs) in the Western Cape, South Africa.

**Methods:**

A qualitative descriptive phenomenological design was used. Participants were recruited by using purposive sampling. Interviews were audiorecorded, transcribed verbatim and analysed.

**Results:**

Three themes emerged from the findings. Theme one: adequate sharing of information with women during the intrapartum period. Theme two: efficient transition of information among midwives and other healthcare providers during the intrapartum period. Theme three: challenges to informational continuity during the intrapartum period.

**Conclusion:**

Communication with the women as well as with other healthcare providers during birth was effective. However, with minimal challenges, informational continuity was effectuated through communication among the midwives, the pregnant women and other healthcare providers.

**Contribution:**

Informational continuity approaches among the midwives, with the women and between healthcare facilities are a prerequisite to ensure continuity of care and care coordination during birth.

## Introduction

Globally, maternal mortality remains a major concern because strategies needed to reduce the maternal mortality rate did not yet meet the desired target of less than 70 per 100 000 live births as targeted by the Sustainable Development Goal (SDG) 3, which focuses on healthy lives for all and the empowerment of women and girls (World Health Organization [WHO] 2018a:7).

South Africa reported approximately 134 maternal deaths per 100 000 live births in 2017 (Dorrington et al. 2019:21). Out of 134 maternal deaths, Western Cape is accounting for 80 deaths at primary healthcare facilities (National Department of Health 2017:10). The distribution of maternal deaths per level of care indicates 31.3% of maternal deaths take place in the maternity obstetric unit (MOU) (National Department of Health 2017:17).

Globally, about half a million women give birth every year (National Department of Health 2017:17). Most of these women are healthy and at low risk of developing complications during labour. In the Western Cape, in Cape Town, these women are cared for in MOUs. Within the MOUs, midwives provide independent care to pregnant women (National Department of Health 2017:17). Care coordination among midwives and care continuity of women during birth require effective information communication approaches among midwives within healthcare settings and with other healthcare professionals.

The intrapartum period is defined as the period from diagnosis of labour to the birth of the baby and includes up to 1 h after the delivery of the placenta (Lowdermilk et al. 2016:376). The WHO has recommended for effective information communication approaches during the intrapartum period as a strategy to reduce maternal morbidity and mortality through the prompt detection, treatment of complications, identification of high-risk women and the rapid referral of women to appropriate levels of care (WHO 2016:1). Informational continuity approaches include patient–provider relationships, collective memory approach, synchronised records and the use of clinical guidelines within the MOUs.

The WHO (2018b:3) has recommended for effective communication during the intrapartum period among midwives and women to ensure for a positive childbirth experience. The midwife should adopt culturally acceptable methods and skills during communication with the women. Midwives are required to engage with women during the intrapartum period and provide women with adequate information concerning their treatment and care, thereby allowing women to actively participate in their care. In a participant observation study performed in South Africa in a public maternity hospital in Limpopo, the findings revealed that effective communication between the women and the midwives had various benefits including enabling women to feel relaxed, confident, special and empowered (Maputle 2018:4). The relationship between the midwife and the women is a pertinent issue during childbirth. Communication should therefore be effective to promote positive relationships with the women (Ahmed 2020:2).

Within primary healthcare (PHC) settings, midwives independently manage and treat women during labour and delivery. They communicate with other members of the multidisciplinary teams among settings should complications arise. Effective communication processes are imperative for the management of women during labour and delivery to ensure early identification of abnormalities and risk factors (M’Rithaa et al. 2017:1). Communication among midwives and with other members of the multidisciplinary team requires up-to-date, clear, timely and comprehensive clinical information in order to achieve cohesiveness within the MOUs (Fealy et al. 2016:20).

The aim of this article was to discuss the experiences of midwives on informational continuity approaches that enabled effective care coordination during birth within the primary healthcare setting. The objectives that are covered in this article were to: (1) explore the experiences of midwives with the positive patient-provider communication approach for effective care coordination during birth, and (2) explore the experiences of midwives with the collective memory approach for effective care coordination during birth.

## Theoretical framework

The authors made use of the Information-Motivation-Behavioural skills (IMB) model as the theoretical framework for this study. The model describes how seeking information motivates a person or group to change behaviour. The information motivates the person or group to develop a set of skills to work towards achieving a set common goal. The person or group with the change in behaviour utilises a set of behavioural skills to work towards a common goal (Fisher, Fisher & Harman 2003:35).

**FIGURE 1:**
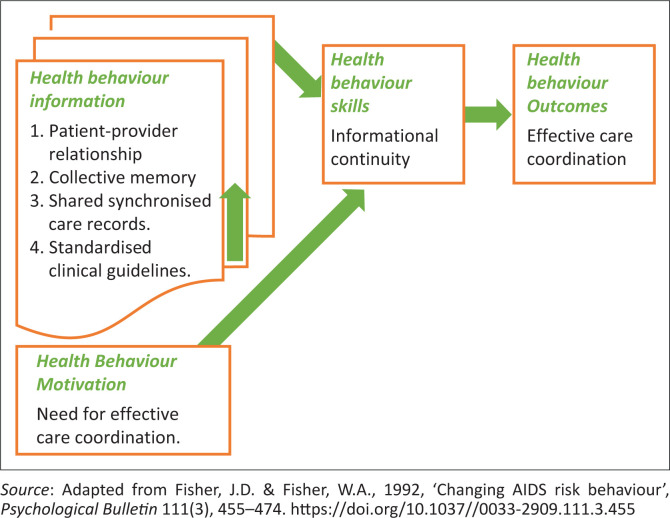
Information-motivation-behavioural theoretical framework.

The IMB model as shown in Figure 1 identifies three constructs, namely health behaviour information, health behaviour motivation and health behaviour skills, which are the factors associated with health behaviour (Fisher et al. 2003:37). All three constructs are needed for successful self-management or adherence to a specific behaviour (Fisher et al. 2003:37).

In this article, the authors only applied the health behaviour information construct of the IMB model. According to the IMB model, *health behaviour information* is defined as the initial requirement for specific health-related behaviour (Fisher et al. 2003:37). The health behaviour information is described in this article as all the information concerning the pregnant women and their condition during the intrapartum period. This information was possible because of the informational continuity approaches, as outlined in Figure 1.

## Research methods and design

A qualitative approach with a descriptive phenomenological study design was used in this study. Husserl’s descriptive phenomenological approach was adopted for the study that emphasises the pure description of an experience (Matua & Van Der Wal 2015:23). Online semi-structured interviews were conducted, transcribed and analysed using Giorgi’s phenomenological approach.

### Research setting

The research was conducted with midwives from two MOUs in Western Cape, South Africa. Western Cape province has two tertiary hospitals for obstetric referrals. Cape Town Metro has four intermediate level referral hospitals and 11 MOUs. The MOUs are exclusively managed by midwives who are telephonically linked with the referral facilities. The researcher performed semi-structured online interviews with the midwives working at two MOUs.

### Population and sampling

Purposive sampling was used to recruit the midwives for this study (Polit & Beck 2018:417). The population comprised all midwives working day and night shift providing maternity care in both Elsies River and Bishop Lavis MOUs who consented to the study. The researcher included all the midwives with a midwifery qualification registered with the South African Nursing Council (SANC) assisting women during the intrapartum period for 6 months and more in the MOUs. All night shift staff declined to partake in the study. All the midwives on annual, maternity and sick leave were excluded from the study.

The sample consisted of 11 midwives working permanently in the MOUs. All the midwives were females. Their experience ranged from 6 months to 18 years. Their ages ranged from 25 years to 48 years of age. The midwives predominantly spoke English and Afrikaans. Four of the midwives were trained in advanced midwifery and neonatology and seven of the midwives were married. The researcher recruited participants at both Elsies River and Bishop Lavis MOUs during Alert Level 3 of coronavirus disease 2019 (COVID-19) with permission from both MOUs’ operational managers, and this was performed by strictly adhering to COVID-19 regulations.

### Data collection

Eleven online interviews were conducted with the use of a semi-structured interview guide with open-ended questions and probing words aligned with each research objective to collect data until data saturation occurred. Data saturation occurred after the 10th interview; the researcher did one more interview and no new data were generated. The participants included five midwives from MOU A and six from MOU B. These interviews explored the experiences of the midwives on informational continuity approaches, which included patient provider relationships, collective memory approach, synchronised care records and the use of clinical guidelines during the intrapartum period.

Interviews were arranged according to a time and date that was convenient for each participant. The author conducted the online interviews with the midwives. Online interviews were used because data collection took place during Alert Level 3 of COVID-19 when physical contact between people was not allowed. Each participant was provided with a data bundle worth R100.00 for the purpose of the online interview. The interviews were conducted in English as this was the predominant language of the participants at both MOUs. Each interview lasted approximately 30 min to 60 min. All interviews were audio-recorded, transcribed verbatim and analysed. The researcher was not known to any of the participants who participated in the study. The researcher had previously received training for conducting interviews at the Stellenbosch University Nursing Department. The researcher attended a workshop where her interviewing skills were assessed in the form of a role-play situation.

The researcher arranged with the managers of both MOUs to have an information session with the participants and to obtain informed consent from participants to participate in the research. Each participant was provided with an information leaflet by the researcher, which explained what the research was about. Both the researcher and the participants adhered to the COVID-19 protocols during the contact session. The participant was given the opportunity to inform the researcher when they will be available for the interview. To test the feasibility of the semi-structured interview guide as the tool for data collection, one pilot interview was performed by the researcher. The pilot interview yielded valuable information and these data were included in the findings of this study.

### Data analysis

Data were analysed by using Giorgi’s (ed. 1985) descriptive phenomenological method of data analysis. The researcher listened and relistened to familiarise herself with the data. The audio recordings were then transcribed verbatim. To understand the meanings of the participants’ experiences, the researcher read the entire transcripts. Significant statements were extracted from the data. Meanings were identified that were relevant to the study to build a coherent structure of experiences. The next step involved the understanding, judgements of relevance and coherent organisation of the constituents of the experiences as described by the participants. The final step involved the grouping together of consistent statements of structures with similar ideas and meanings to identify themes and sub-themes, which described the lived experiences of the participants.

### Measures of trustworthiness

To ensure trustworthiness, the criteria of Guba and Lincoln 1985 as cited by Cypress (2017:256) were used and include credibility, transferability, dependability and confirmability. To ensure credibility, member checking was performed by taking the findings back to the participants for verification, and the participants agreed with them and did not remove or add any new information.

Transferability was ensured by using purposive sampling, ensuring that participants could provide detailed specific information about the context. Dependability was ensured by making sure that all the steps of the research process were well documented. Confirmability was ensured by sending the audio recordings and transcripts to the study supervisor for verification.

The researcher previously worked as a student midwife in MOUs and is now working as a midwife in a district hospital. The researcher therefore had preconceived ideas about how communication took place within MOUs between pregnant women and midwives as well as among the members of the multidisciplinary teams and with referral hospitals. The researcher was also aware of some of the challenges with communication. The researcher used a reflective journal to purposefully reflect and wrote down her prejudices and presumptions regarding the topic under investigation.

Continuous bracketing was performed by the researcher throughout data collection and analysis to avoid preconceptions of the researcher to interfere with the final descriptions of the experiences of the midwives in order for the researcher to attend to the phenomenon in an unprejudiced approach (Grove, Gray & Burns 2015:69).

### Ethical considerations

Ethical approval was obtained prior to commencement of the study from the Stellenbosch University Health Research Ethics Committee in October 2020 (reference: S20/08/192). Permission was obtained from the Western Cape Department of Health to conduct this study at the Elsies River and Bishop Lavis MOUs. Institutional permission was requested from both MOUs before the study commenced (reference: WC-202101 002). Ethical considerations observed during data collection were the right to confidentiality, self-determination, protection from harm and anonymity. Self-determination was ensured by the researcher by providing each participant with adequate information regarding the study in the form of an information leaflet. The participants could comprehend the information. They were given the freedom to decide whether they wanted to take part in the study or not. Confidentiality was ensured by assigning a code in the form of a number to each participant and ensuring that the audio recordings and transcripts did not reflect any personal details of the participants. This ensured anonymity throughout data collection and data analysis and in the research report. Participants were protected from harm and discomfort by informing them that they could withdraw from the study at any time if they experienced discomfort during participation and by encouraging them to verbalise any such discomfort.

Before commencement of the online interviews, written consent to share participants’ contact details with the researcher was obtained. Consent to share their contact details was included in the consent form to participate in the study. The contact details were used to provide each midwife with a R100.00 data bundle for the purpose of the online interview and a separate data bundle for personal use to the value of R100.00 as reimbursement for their time.

## Results

Three themes emerged from the findings. Theme one: adequate sharing of information with women during the intrapartum period. Theme two: efficient transition of information among the midwives and with other healthcare providers during the intrapartum period. Theme three: challenges to informational continuity during the intrapartum period.

### Theme 1: Experiences with adequate sharing of information with women during the intrapartum period

The subthemes for this theme were the experiences of intention of communication between the midwives and pregnant women and the communication skills of midwives.

#### The experiences of intention of communication between the midwives and pregnant women

The midwives provided various reasons for their intention to communicate with the women. The midwives indicated that they provided the women with prompt communication from the time of admission in the MOUs. The midwives expressed that they shared information with the women, and at the same time, they sought information from the women during communication:

‘On initial contact with the woman, we will start to chat and get to know one another. I will share information with the woman and explain information to her regarding the birth process.’ (Participant 2, female, advanced midwife, 42 years old)

The midwives indicated that while communicating with the women they were able to build a trust relationship, which allowed the women to be less anxious, increased their confidence, which allowed conversation and they were involved in decision making:

‘Firstly, communication contributes to building trusting relationships between me and the women during birth, if there is trust the women will trust me as the midwife to make decisions concerning her management during birth.’ (Participant 1, female, midwife, 32 years old)

‘Talking with the women increased the women’s confidence and they became more willing to share information.’ (Participant 4, female, midwife, 27 years old)

#### The communication skills of midwives

The midwives in the study asserted that they adopted various verbal and non-verbal communication skills while communicating with women during birth. The midwives explained that they communicated with the women in a language that the women understood. The midwives in the study predominantly spoke English and Afrikaans. The midwives experienced that if they used a language that the women understood, the women became more willing to communicate with them:

‘We interact with the women and try to speak in a language that the women understand, this improved communication since they understood the conversations.’ (Participant 5, female, advanced midwife, 35 years old).

‘I will also calm her by making eye contact with her, if my body language and attitude is calm my patient will be calm.’ (Participant 2, female, advanced midwife, 42 years old).

The midwives used verbal and non-verbal communication skills during interactions with the women. The midwives explained how the tone of their voices during communication with the women decreased anxiety and made the women calm:

‘If you are calm and speak in a soft but audible tone of voice with the women, the women become calmer and they appear to be less anxious.’ (Participant 5, female, advanced midwife, 35 years old)

### Theme 2: Experiences with efficient transition of information among the midwives and other healthcare providers during the intrapartum period

The subthemes under this theme included the importance of communication among midwives, the methods of communication among midwives and other healthcare providers during birth, and the documents used to communicate during birth.

#### Experiences with communication among midwives within the maternity obstetric units

Sharing information among midwives was expressed as vital. The midwives mentioned that sharing information contributed to effective care coordination among the midwives and this enabled the provision of care as per the specific needs of the women as well as ensuring safe maternity care:

‘We share the responsibility to take care of women. We need to be informed because we share the responsibility to provide care to the women. This allows us to follow up on care and to ensure care is continuous.’ (Participant 10, female, midwife, 30 years old)

‘Sharing information with the other midwives is important. Having all information regarding women during birth enables you as the midwife to provide safe maternity care.’ (Participant 5, female, advanced midwife, 35 years old)

#### The methods of communication among midwives and other healthcare providers during birth

Different methods of communication were utilised by the midwives within the MOUs and with other healthcare providers at the transfer facilities. This included the use of the maternity case record (MCR) and verbal communication that included telephonic communication and communication during handover rounds and staff meetings:

‘We use information recorded in the in the MCR to communicate information among us as midwives to ensure that all midwives have the same information regarding the women.’ (Participant 11, female, advanced midwife, 48 years old)

‘We phone the midwives and obstetricians at the referring facilities. This information that we share will assist them to continue the care as needed by the women upon arrival.’ (Participant 9, female, advanced midwife, 40 years old)

‘We will have regular meetings during our shifts. We use these meetings to discuss management strategies as well as plans for management of women in the MOUs.’ (Participant 6, female, midwife, 25 years old)

Communication among the midwives in the MOUs occurred during handover rounds. The handover round took place at shift changes and during breaks:

‘We hand over to one another. This handover will include all relevant information concerning a woman’s progress during birth.’ (Participant 3, female, midwife, 36 years old)

‘Between shifts we will have a handover round from bed to bed to ensure the care of the women is continuous.’ (Participant 5, female, advanced midwife, 35 years old)

#### Documents used to communicate during birth

Different documents are used to exchange information during the intrapartum period. The midwives used the partogram to share information regarding the women’s condition and the progress of labour. The midwives explained that when they transferred the women, they completed the ISBAR tool. The ISBAR tool is a clinical report on a woman’s maternity situation and is filled in duplicate within the MCR:

‘We use the partogram to document both maternal and foetal observations. This document is used by us to share information among us as midwives.’ (Participant 5, female, advanced midwife, 35 years old)

‘We make use of the ISBAR form, which is the identify, situation, background, assessment, and recommendation sheet, which includes information concerning both maternal and foetal observations. The document is valuable when emergencies arise, and during transfers to share information with other midwives or obstetricians at the transferring facilities.’ (Participant 1, female, midwife, 32 years old)

### Theme 3: Challenges experienced to informational continuity during the intrapartum period

The theme will be discussed under four sub-themes: (1) The language challenges experienced in communication with pregnant women, (2) pregnant women not bringing MCR to the MOU, (3) the challenges of communication among midwives, and (4) challenges with clinical guidelines.

#### The language challenges experienced in communication with pregnant women

Various challenges in communication are experienced by the midwives in the MOUs. The challenges included women from various foreign countries who only spoke their language and some women who came to the MOU without their MCRs:

‘Communication becomes difficult for me if the women speak a foreign language. I try to communicate but can clearly see that the women do not understand what I am trying to say to her.’ (Participant 5, female, advanced midwife, 35 years old)

Midwives experienced that the pregnant women from foreign countries, such as Somalian women did not understand the main languages of communication predominantly used in the MOUs, namely English, Afrikaans and isiXhosa. This made it difficult for the midwives to instruct, update and support the Somalian women during labour:

‘We try our best to communicate with the women, but if they do not understand our language, we are unable to explain to them what is expected of them.’ (Participant 1, female, midwife, 32 years old)

#### Pregnant women not bringing maternity case record to the maternity obstetric unit

Some of the women came to the MOU without their MCRs. The midwives stated that this resulted in duplication of interventions and fragmentation of care for the women because of missing information:

‘The women sometimes come to the MOU without their maternal case records, and we will have to do all the booking tests again, this makes it difficult because we will have no information.’ (Participant 9, female, advanced midwife, 40 years old)

#### The challenges of communication among midwives

It emerged that the midwives experienced challenges with communication, which included incomplete documentation, inaccessible records and inadequate communication during handovers and referrals:

‘Sometimes the documentation in the women’s maternity case record is incomplete. This makes it difficult to follow up on the women’s treatment and care.’ (Participant 9, female, advanced midwife, 40 years old)

‘They are in a hurry when handing over to catch their transport and sometimes forget to share important information regarding the women.’ (Participant 6, female, midwife, 25 years old)

The midwives identified incomplete documentation and incomplete information recorded during birth as a challenge with communication. The midwives stated that this might result in duplication of interventions:

‘They will perform vaginal examination and will not record this information on the partogram. This results in duplication of vaginal examinations which causes discomfort for the women and puts the women at risk of developing infections.’ (Participant 4, female, midwife, 27 years old)

The midwives experienced difficulties with documentation because more than one midwife provided care to the women in the MOUs. One midwife might be busy documenting while the other might be busy observing the woman and will not have the MCR to record the findings. According to the midwives, the findings were sometimes recorded on a separate page to be recorded later, which led to information not being recorded:

‘If another midwife is busy with the woman’s maternity case record, we record the information on a separate note and will record the information on a later stage, but if the MOU gets busy you can easily forget to record this information, and this may lead to miscommunication and errors in record keeping.’ (Participant 8, female, midwife, 38 years old)

The midwives provided maternity care to several women at the same time, and sometimes the MOUs became very busy, which resulted in MCRs being misplaced or even mixed up among women’s files:

‘If the MOU gets busy, the maternity case records get mixed up or sometimes even misplaced, since we as midwives provide care to several women at the same time.’ (Participant 2, female, advanced midwife, 42 years old)

#### Challenges with clinical guidelines

The midwives mentioned that they experience certain challenges with clinical guidelines which included the fact that they were not part of the development of these guidelines.

## Discussion

**FIGURE 2:**
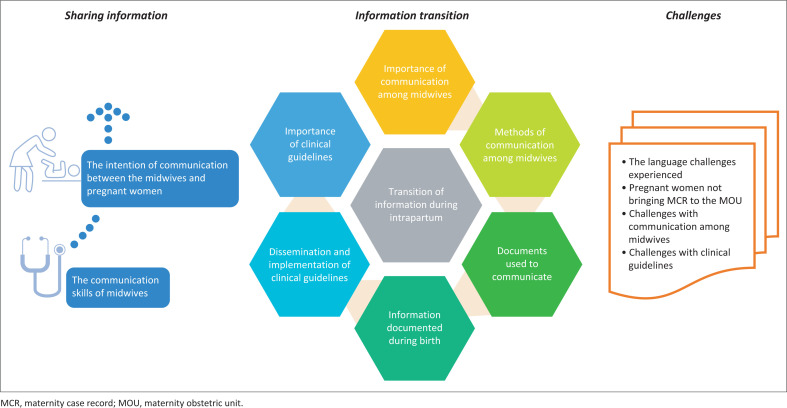
A summary of findings.

Figure 2 summarises the key findings of this study. The study revealed that sharing of information and the transition of information during the intrapartum period were crucial aspects in enabling informational continuity for effective care coordination during birth in the MOUs in the Western Cape.

The research question for this study was this: what are the experiences of the midwives on informational continuity during birth within the PHC settings in the Western Cape. The study’s findings revealed that informational continuity during birth enabled effective care coordination among the midwives. Communication during birth was described as an important element in providing quality maternity care within the MOUs. Communication was experienced by the midwives as a continuous process among themselves within the MOUs and with other members of the multidisciplinary team. However, the midwives experienced various challenges to communication, which included language barriers, incomplete documentation, and inaccessible records.

The WHO describes the relationship between pregnant women and midwives as a positive patient-provider relationship (WHO 2018a:18). Patient-provider communication refers to the shared and mutual understanding regarding information among the midwives and the pregnant women in the MOUs. Effective communication took place between the midwives and the women during the intrapartum period. This included sharing information with the women regarding their care required and in certain instances why their care is changing. The midwife is a key component of labour and birth. They provide the women with the necessary support and assist the pregnant women safely through childbirth.

Effective communication between pregnant women and midwives is an important factor in the women’s satisfaction and the provision of safe maternity care (Ahmed 2020:2). World Health Organization recommended effective communication using culturally accepted methods and skills during communication between women and midwives during labour to ensure for a positive childbirth experience (WHO 2018b:3). It is a pertinent issue for a positive relationship to be established between the pregnant woman and the midwife during childbirth. Therefore, for the midwife to fulfil this role, communication should be effective with the women to ensure for a positive relationship between the woman and the midwife (Ahmed 2020:2).

The study revealed that the midwives made use of verbal communication skills during communication with the women in the MOUs. This included speaking clearly and in a soft but audible tone of voice during interactions with the women. This finding is similar to that of a study performed in Limpopo province, South Africa, to explore and describe the experiences of midwives regarding the management of women in labour, in which it was found that midwives were open and supportive towards women through verbal communication and that they listened to the women in order to understand their unique wishes and differences (Maputle & Hiss 2010:9).

In this study, all the midwives who participated in this study experienced communication challenges with women from foreign countries, such as Somalian women, who did not understand the main languages of communication predominantly used in the MOUs, namely English, Afrikaans and isiXhosa. This made it challenging for the midwives to instruct, update and support the Somalian women during labour. This finding is supported in an exploratory descriptive study carried out in Limpopo province in a hospital that provided maternity care to different racial groups and in which language barriers were found to be a challenge to effective communication with the women. The midwives found it difficult to communicate with the women from foreign countries that were transferred to the hospital if the women spoke a language different from that of the midwives (Maputle & Hiss 2010:10).

Women not bringing their MCR along to the MOU visit, was another challenge to communication. One of the interventions to improve maternity care for pregnant women was the introduction of the MCR. The MCR in 2010 was one of the interventions to improve maternity care of pregnant women. The MCR includes all information regarding the woman’s current pregnancy and obstetric history. This is a client-held record and an official tool of communication between the different levels of care and health facilities. Each pregnant woman presenting at the healthcare facility should receive an MCR that should be completed at each visit (National Department of Health 2015:29–30). Most of the midwives in this study revealed that the women at times forgot to bring their MCRs along to the MOUs upon arrival in labour. The midwives mentioned that this was very time consuming for them because they had to repeat all booking tests and history taking, which ultimately affected the continuity of care for the women in the MOUs by delaying care.

According to the IMB model, the health information behaviour construct refers to the patient-provider relationship (Fisher et al. 2003:37–38). According to the IMB model, the patient–provider relationship is the health information behaviour that motivated the midwives to adopt the skills necessary to effectively communicate with women during birth. The skills necessary to ensure a positive patient–provider relationship included positive attitudes, appropriate communication skills and professionalism. These skills were portrayed by the midwives in this study to effectively communicate with women during birth. Hence, this study’s findings indicated that midwives had positive patient–provider relationships with women during birth.

The midwives described the various verbal and nonverbal communication skills adopted by them during communication with the women in the MOUs. The midwives mentioned that they respected the women’s cultural differences by using a language that the women understood when they communicated with the women. The midwives provided services to a diversity of women. This included women who predominantly spoke Afrikaans, English and isiXhosa and women from Somalia. According to the standards of proficiency for midwives published in 2019 by the Nursing and Midwifery Council, Domain 6 refers to all the midwifery skills needed during communication (Nursing and Midwifery Council 2019:33). The skills for communicating with women should consider a woman’s needs, views and preferences. The midwife should effectively listen and respond to verbal and nonverbal cues and make use of positive verbal and nonverbal reinforcement.

Collective memory refers to the sharing of knowledge and communication of information among the midwives regarding pregnant women in the MOUs and with other healthcare providers at referral facilities to ensure that all midwives and other healthcare providers have the same information regarding the women in their care (WHO 2018a:18). Communication involves communication among the midwives in the MOUs and with other healthcare providers at transfer facilities. In the MOUs, the midwives adopted various methods of communication.

Transition of information was a crucial component among the midwives within the MOUs and with other healthcare providers at the transferring facilities. This improved care coordination among the midwives in the MOUs and other healthcare providers, which ultimately ensured continuity of care as well as the provision of safe care for the women during the intrapartum period.

The midwives in the MOUs communicated among each other within the MOUs during handover rounds, through documentation, which included the ISBAR form, the MCR and the partogram. They also communicated telephonically and with documents, which included the MCR and the ISBAR form with the healthcare providers at the transferring facilities during transfer of the women.

Communication among healthcare providers in all care settings is classified as one of the approaches identified for optimising care coordination in healthcare settings (WHO 2018a:18). The midwives in this study relayed the importance of communication among themselves in the MOUs and with other healthcare providers at transfer facilities. During the interviews, all the midwifes said that they had a shared responsibility to provide care to the pregnant women in the MOUs and that they therefore communicated while they provided care to the women through handovers to allow for the care of the women to continue and to facilitate care coordination among the midwives in the MOUs and healthcare providers at transfer facilities. This finding is similar to a study on informational continuity and collaboration among healthcare providers in Australia, which were found to be of paramount importance for the provision of safe maternity care and to ensure care coordination and continuity of care for women during birth (Downe, Finlayson & Fleming 2010:250).

All midwives mentioned that communication between them and other healthcare providers at transfer facilities assisted them with decision making regarding a woman’s management and care. According to Schwartz, Lowe and Sinclair (2010:1), information communication within and among healthcare settings is essential to success in the work setting. The study by Schwartz concluded that informational continuity was needed to allow midwives to make important healthcare decisions in the best interest for both the pregnant woman and her unborn baby in order to promote their health and well-being.

Effective communication among midwives was achieved through verbal communication in the form of handover rounds that occurred face to face with all midwives present. All the midwives described how they communicated with each other in the MOUs at the start and at the end of their shifts during handover rounds. One midwife said that they went from bed to bed to discuss the women’s management plans and their progress of labour.

Some of the midwives further elaborated and said that the handover rounds ensured that they all had the same information concerning the women’s treatment and care to ensure care continuity for the women and care coordination among the midwives and other healthcare providers. Similarly, a study performed in South Africa in the Western Province, found that handovers played a critical role in the treatment and care of women during the intrapartum period. The handover process was the entry point for the exchange of information among the midwives who assisted midwives with gaining insight into the care of the women in the MOUs (M’Rithaa et al. 2015:4).

The midwives in this study mentioned that they were telephonically linked to the referral facilities. All the midwives made telephonic contact with the obstetricians at the referral facilities during transfer of the women or if they required guidance on how to manage the women if complications arose in the MOUs. One midwife elaborated and stated that they would telephonically discuss the women and share all information relevant with the transfer facilities to follow up on the women’s care. The findings are supported by M’Rithaa et al. (2015:4–5) who also found that midwives in the MOUs relied on phone calls for consultation if they were unsure of how to proceed and if women needed to be referred as there were no doctors in the MOUs.

The midwives also revealed that they communicated during staff meetings where they would provide feedback to each other if they had gone for training or workshops. They would also discuss the management of the women if complications arose to plan for the referral of the women. Care coordination of the pregnant women within and among healthcare settings could be facilitated by effective communication among midwives within the MOUs and with other healthcare providers. The WHO (2018a:20) recommends for all healthcare providers to work as a team and effectively communicate among each other as this will ensure care coordination and care continuity for pregnant women within and among healthcare settings. According to this study, continuity of care and care coordination for pregnant women were achieved through effective communication among midwives in the MOUs and with other healthcare providers.

The midwives, however, mentioned the incomplete documentation in the MCR and said that this made it difficult for them to follow up on a woman’s care.

One midwife said that midwives sometimes forgot to record interventions performed with women during the intrapartum period, which often resulted in duplication of interventions and unnecessary discomfort for the women. This finding is supported in a study performed in two maternity hospitals in Germany by Lippke et al. (2019:1) during which it was noticed that ineffective communication because of incomplete record keeping was a major contributing factor in duplication of interventions and adverse events in maternity care. Similarly, a descriptive cross-sectional study was carried out in South Africa to determine the existing data and to provide a report on the performance of PHC facilities (Bresick, Von Pressentin & Mash 2019:111). The study found that ineffective informational continuity contributed to fragmentation of care and a lack of care coordination for pregnant women (Bresick et al. 2019:114).

Another challenge in communication among the midwives in the MOUs was inaccessible records. Most of the midwives complained that the MOUs often became very busy and that during this time, MCRs often were misplaced or mixed up among women’s files. The midwives would make use of a separate page to document interventions and findings and put this aside to document at a later stage. This often resulted in record keeping not being performed, leading to misinformation. M’Rithaa et al. (2015:5) also found that incomplete documentation and documentation not performed at all resulted in ineffective communication that could be time consuming and could result in serious adverse events. This also resulted in challenges when women were transferred from MOUs to referral hospitals.

The health information behaviour in this study was the collective memory approach among the midwives in the MOUs and with other healthcare providers at transfer facilities, which according to the midwives in this study motivated them to share information regarding the women among themselves in the MOUs and with other healthcare providers at transfer facilities. The sharing of information ensured that all midwives had the same information regarding the women, thus ensuring the collective memory approach among the midwives and other healthcare providers at transfer facilities. For the midwives in this study, the sharing of information among themselves in the MOUs and with other healthcare providers at transfer facilities had various benefits, including the continuation of care for the pregnant woman, care coordination between the midwives and other healthcare professionals, prevention of negative incidents and informed decisions regarding a woman’s care, allowing for the provision of care specific to the needs of the women and the provision of safe maternity care within the MOUs.

The synchronised care records used by the midwives in the MOUs included the partogram, a tool of communication that the midwives used to record information pertaining to the progress of labour and the management of the women during birth. The ISBAR tool was used within and among facilities to provide a detailed summary of the women’s condition and reason for transfer and admission. The documents are all part of the MCR. The Maternity Care Guidelines in South Africa (National Department of Health 2015) recommend for the use of a standardised MCR by all birthing facilities in order to improve the quality of maternity care provided to pregnant women (Sibiya, Cele & Ngxongo 2015:53).

In this study, the midwives stated that they were familiar with the use of the MCR. One of the midwives said that they used the MCR to seek information regarding the care of pregnant women and to document the care provided to women during birth. Another midwife also stated that the MCR assisted them with communication through documentation of information among themselves as midwives and improved the quality of care of pregnant women. Sibiya et al. (2015:53) supported this finding and found that the use of the standardised MCR improved the quality of care and care continuity of women during pregnancy, the intrapartum and the postpartum period.

### Strengths and limitations

The descriptive phenomenological method allowed for the author to perform in-depth interviews and to extract meaningful data from the participants, which helped to describe their experiences. Analysis of the data using Giorgi’s (ed. 1985) phenomenological analytical approach assisted in providing an in-depth description of the experiences of the midwives. The researcher made use of member checking. The researcher returned the collected data in the form of transcripts and audio tapes to the participants and asked them to verify the data to ensure that the data collected during the initial interviews was a true reflection of their experiences.

The study was performed only at two MOUs in the Western Cape. The study sample was a relatively small sample size of the study population and affected the transferability of the research findings to other MOU settings. The researcher requested permission to do research at another MOU but was denied permission because the MOU already accommodated multiple other students doing their research at the institution. However, the data from the interviews provided an understanding of the midwives’ experiences regarding informational continuity approaches during birth to enable effective care coordination during birth within the MOUs.

The midwives who were interviewed were all working day shift, and the findings of the study cannot therefore be generalised to the midwives working night shift. The researcher approached the midwives on the night shift, but they all declined to participate in this study.

### Implications and recommendations

The midwives experienced communication challenges with women of foreign nationalities. The facilities should allow the women to bring their own interpreter with them when coming to the MOUs. According to the midwives, the guidelines were only part of a discussion by the operational manager, hence regular training should be provided on new clinical guidelines.

## Conclusion

The findings of the study revealed that informational continuity was achieved through effective communication with the pregnant women during the intrapartum period in the MOUs. Communication was continuous between the midwives and the women and contributed to building the women’s confidence, involving women in their care, decreased anxiety, facilitated cooperation, and allowed women to make informed decision concerning their care during birth. Challenges in communication among midwives and women identified included language barriers. This was specifically with women from foreign countries. Another challenge was that women sometimes forget to bring their MCR to the MOUs and this was time consuming for the midwives because this resulted in duplication of care interventions and fragmented care for the women during the intrapartum period.

Informational continuity was achieved through communication among the midwives in the MOUs and with other healthcare providers at transferring facilities. Communication among the midwives and other healthcare providers was important to the midwives in the MOUs because it facilitated care coordination and continuity of care for the women during birth. The communication among the midwives and other healthcare providers was effective and assisted midwives with decision making, prevented negative incidence and promoted the provision of quality and safe maternal care in the MOUs. Communication among the midwives and other healthcare providers mainly occurs through written communication in the MCR, the partogram and the ISBAR tool. Midwives also communicated verbally during handover round, telephonically and during staff meetings. Challenges with communication among midwives included incomplete documentation, inaccessible records, and inadequate communication during handovers.

The midwives used the intrapartum care guidelines from WHO and the South African guidelines on intrapartum care to guide their care provided to women in the MOUs. The guidelines ensured standardised maternity care within the MOUs. Midwives, however, need more training when new guidelines become available.

Therefore, informational continuity in the MOUs was achieved through the four informational continuity approaches, which include positive patient provider communication, the collective memory approach among midwives, the use of synchronised care records, and the use of standardised clinical guidelines. Effective informational continuity within the MOUs could allow for care coordination and continuity of care during the intrapartum period.
